# Betulin-3,28-diphosphate as a Component of Combination Cytostatic Drugs for the Treatment of Ehrlich Ascites Carcinoma In Vitro and In Vivo Experiments

**DOI:** 10.3390/scipharm86020017

**Published:** 2018-04-23

**Authors:** Olga Vorobyova, Olga Deryabina, Darina Malygina, Nadezhda Plotnikova, Anna Solovyeva, Kseniya Belyaeva, Nina Melnikova

**Affiliations:** 1 Department of Pharmaceutical Chemistry, Federal State Budgetary Educational Institution of Higher Education “Privolzhsky Research Medical University”, Ministry of Health of the Russian Federation, Minin sq., 10/1, 603005 Nizhny Novgorod, Russia; vorobeva_olga1990@mail.ru (O.V.); mds73@yandex.ru (D.M.); sannag5@mail.ru (A.S.); kseniyabelyaeva2018@bk.ru (K.B.); 2 Department of Chemistry, N.P. Ogarev Mordovian State University, Bolshevistskaya St. 68, 430005 Saransk, Russia; dr.deryabina@gmail.com (O.D.); plona@mail.ru (N.P.)

**Keywords:** Ehrlich carcinoma, betulin-3,28-diphosphate, 5-fluorouracil, hydrazine sulfate, antioxidant activity

## Abstract

The activity of betulin-3,28-diphosphate (BDP) in combination with the cytostatics such as 5-fluorouracil (5-FU) and hydrazine sulfate (HS) was demonstrated by using the transplanted Ehrlich ascites carcinoma (EAC) in mice. The dose-dependent effect of combination drugs BDP + HS and BDP + 5-FU was revealed by in vitro experiments on rats. The synergetic effect of HS and BDP on oxidative stress and energy metabolism was established. The malonic dialdehyde (MDA) level both in plasma and erythrocytes decreased by 87 ± 2%, and the superoxide dismutase (SOD) activity increased by 105 ± 7% in comparison with the control. The combination of BDP + HS promoted the increase of lactate dehydrogenase (LDH) activity in the reverse reaction by 195 ± 21% compared to the control. The combination drug of 5-FU with BDP caused the synergetic decrease of the lipid peroxidation (LPO) intensity estimated by the MDA level decrease up to 14 ± 4% compared to pure compounds. Betulin-3,28-diphosphate in combination with cytostatics for EAC treatment improved the animal health status, as well as decreased the cytostatics dose that can be used in palliative therapy.

## 1. Introduction

Patients’ quality of life is an important issue in oncology in parallel with optimal pharmacotherapy by cytostatics, radiation therapy, and/or surgical interventions. The use of modern highly effective cytostatics has adverse side effects and a general toxic effect on the body [1,2,3].

The toxic effects of cytostatics are largely determined by their bioavailability, which depends on the hydrophilic-lipophilic ratio and solubility of drugs. As a rule, highly water-soluble substances have severe systemic toxic effects. The modern trend of pharmacotherapy is to use well-proven drugs in new dosage forms or new methods of administration, such as selective delivery when the dose of cytostatics can be dramatically reduced [4,5,6]. The inexpensive cytostatics such as hydrazine sulfate (HS), Sehydrin and 5-fluorouracil (5-FU), characterized by a low median lethal dose (LD_50_) value close to 0.32 mg/kg, are of interest as a component of a new dosage form in clinical practice [7,8]. 5-fluorouracil inhibits thymidylate synthetase, disrupts the nucleic acids synthesis, and leads to the apoptosis of tumor cells. 5-fluorouracil is used to treat of human hepatocellular and esophageal carcinoma, colorectal and pancreatic cancer, etc. [9,10,11,12]. Hydrazine sulfate is an insufficiently explored antitumor agent, well-proven in the treatment of tumors of the liver, lungs, mammary glands, intestines, and cervix [13,14,15]. The mechanism of HS action includes both the competitive inhibition of monoamine oxidase, leading to the increase of biogenic amines and amino acids (dopamine and serotonin) levels, and the blocking of gluconeogenesis by phosphoenol-pyruvate carboxylase [16].

Natural compounds are capable of interacting with cytostatics in synergy and may be another component of a dosage form leading to the cytostatic dose reduction [17]. The synergistic effect of 5-FU with natural compounds, such as polyphenols, triterpenoids (betulinic, ursolic, and oleanolic acids), and flavonoids (flavone and catechin) has been shown in the papers [9,10,11,12,18,19]. Triterpenoid acids (ursolic, betulinic and oleanolic acids) providing a complex action with other antitumor drugs, e.g., irinotecan and cisplatin, were studied in detail. The feature of natural compounds action is a stronger synergetic effect of extracts enriched with the mixture of triterpenes and flavonoids, for example, the extract of Japanese apricot, rather than pure triterpenic acids [7].

Betulin-3,28-diphosphate (BDP) as a component of combination drugs was chosen due to its ability to act as a complement inhibitor, this being an important property of antitumor agents [20]. Betulin and its derivatives such as betulinic and betulonic acids, their esters, and amides have a cytotoxic effect on tumor cells and a low toxicity with respect to nontumor cells. The antitumor activity of these compounds has been shown by in vitro and in vivo experiments in the treatment of melanoma, neuroblastoma, hepatoma, etc. [21,22]. Antioxidant properties play an important role in achieving an antitumor effect.

It has been reported recently that natural antioxidants, such as β-carotene, honokiol, and curcumin, may be used in a novel anticancer therapeutic strategy by combining them with 5-FU [23,24,25]. The combination of 5-FU and β-carotene showed greater tumor growth inhibition in an Eca109 xenograft mouse model than a single agent with a low systemic toxicity. The studies demonstrated that the combined administration of 5-FU and β-carotene significantly downregulated the protein levels of Cav-1, p-AKT, p-NF-κB, p-mTOR, p-p70S6K in Eca109 cells more effectively than 5-FU alone [23]. Honokiol and 5-FU exerted a synergistic therapeutic effect on oral squamous cell carcinoma by inducing apoptosis, and this combination enhanced the efficacy without increasing 5-FU toxicity [24]. Curcumin combined with 5-FU led to lower cell viability and higher apoptosis than 5-FU administered alone in human esophageal squamous cell carcinoma [25].

**Figure 1:**
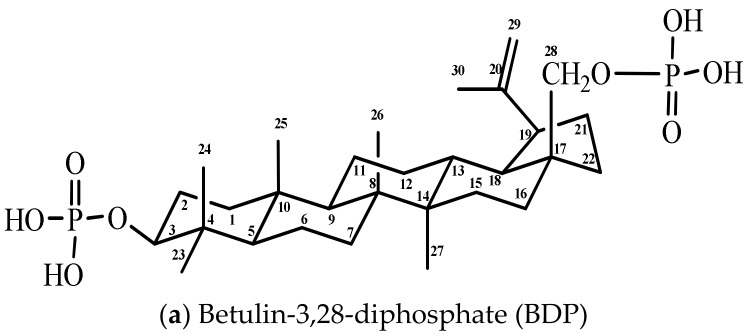

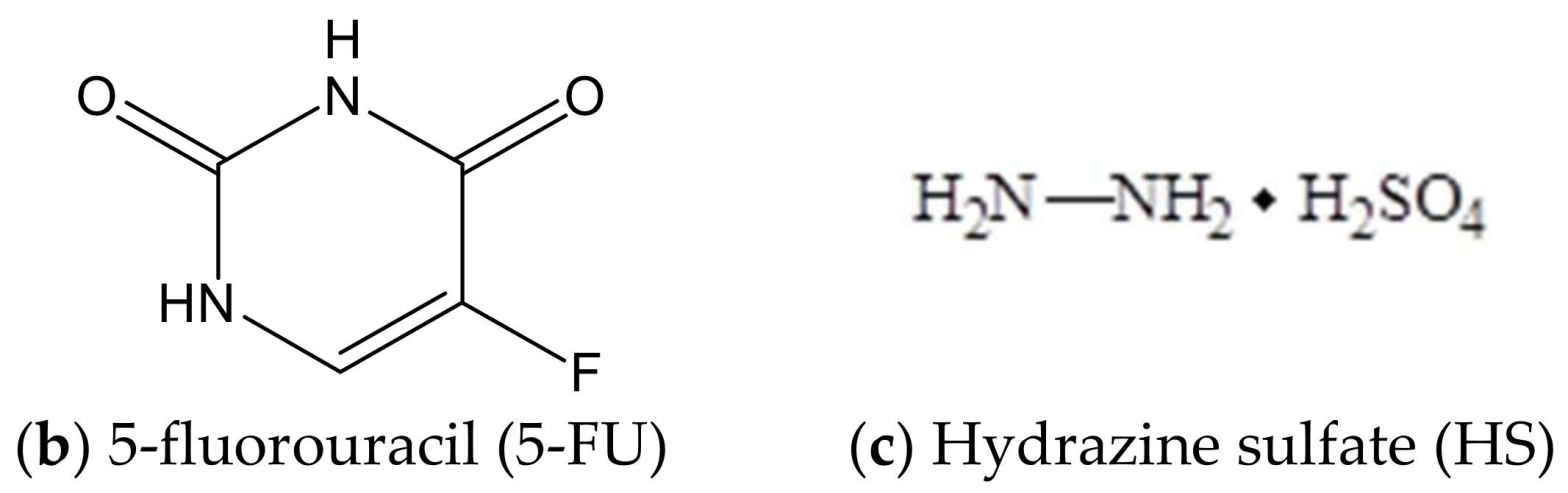
Formulas of components of combination drugs

The present work has studied the effect of combination drugs of 5-fluorouracil and hydrazine sulfate with water-soluble betulin-3,28-diphosphate (Figure 1) shown by in vitro experiments on rat blood and by using the transplanted Ehrlich ascites carcinoma model in mice. The antioxidant properties and the dose-dependent effects of combination drugs were studied using the biochemical indexes such as superoxide dismutase (SOD) activity, malonic dialdehyde (MDA) level in plasma, and erythrocytes in rat blood. Energetic metabolism was determined by lactate dehydrogenase (LDH) activity in direct and reverse reactions. The characteristics of the animal health status, such as survival, body weight, wool, behavior, and reduction in cardiotoxicity, have been revealed by experiments on mice.

## 2. Materials and Methods

### 2.1. Materials

Betulin was isolated from Betula Pendula bark using the methods in [26]. 5-fluorouracil (Sigma Aldrich, Moscow, Russia), hydrazine sulfate (Sigma Aldrich), purified water (resistivity ≥18 MΩ·cm, Millipore, Merck, Darmstadt, Germany).

### 2.2. Synthesis of Betulin-3,28-diphosphate

Betulin-3,28-diphosphate was synthesized according to the methods in [27]. Betulin (6.0 g, 13.56 mmol) was solved in the mixture of 120 mL of dioxane and 7.08 mL of pyridine (81.6 mmol) in a three-neck flask at room temperature. Phosphoryl chloride (POCl_3_) (7.56 mL, 81.6 mmol) in 60 mL of dioxane was added to the betulin solution dropwise into the flask at control temperature (up to 25 °C). Then, the reaction mixture was stirred for 24 h. 1000 g of a water and ice mixture was added to the reaction mixture, and then the white precipitate was filtered and washed several times with water. The wet sediment containing 25% water was isolated that corresponded to betulin-3,28-diphosphate hydrate (betulin-3,28-diphosphate·xH_2_O, where x = 8–9). Infrared (IR)-spectrum (KBr, cm^−1^): 3421 broad (brd.) (ν OH), 1641 wk. (ν P=O, ν C=C), 1240 brd. (*δ* P=O), 1031 brd., 973 brd. (ν C–O), 501 nw. (ν P-O). The BDP carbon-13 nuclear magnetic resonance (^13^C-NMR) spectrum (tetramethylsilane (TMS), DMSO-*d*_6_, *δ*, ppm (delta shift), Table 1).

The BDP ^1^H-NMR spectrum (TMS, DMSO-*d*_6_, *δ*, ppm): 0.68–1.99 (42H, 6CH_3_, (CH_2_)n. m.), 2.35 (1H at C19, two t.), 3.70 (1H at C3, = CHOP, two t.), 3.95 and 3.52 (2H at C28, CH_2_OP, two d.), 4.55, 4.68 (2H at C29, CH_2_=C, 2 s.), 5.72 (protons in phosphate groups O–P(OH)_2_, broad diffuse s.).

Infrared-spectra were recorded on “IR Prestige-21” (Shimadzu, Kyoto, Japan) in the range of 4000–500 cm^−1^ (KBr pellet). Reversed phase-high-pressure liquid chromatography (RP-HPLC) analysis were carried out on “LC-20Avp” (Shimadzu, Kyoto, Japan) with ultra violet-detection, column is Discovery C18 (25 cm × 4.6 mm, 5 μm, Supelco, Merck), retention time τ was equal to 5.19 min.^13^C, ^1^H, ^31^P-NMR spectra were recorded using NMR-spectrometer “JNM-ECX400” (Jeol, Kyoto, Japan), DMSO-*d*_6_, CDCl_3_, D_2_O; 101, 400 and 202.46 MHz, respectively.

The sodium salt of BDP was obtained by dissolving of BDP in alkaline solutions of Na_2_CO_3_ or NaOH.

### 2.3. Biological Activity

Male Wistar rats (200–250 g) and male mice (20–30 g) were involved in the study. The animals were purchased from the Animal Breeding Facilities “Stolbovaya” (Chekhov, Moscow region, Russia). All procedures for maintenance and sacrifice (care and use) of animals were carried out according to the criteria outlined by European Convention ET/S 129, 1986 and directives 86/609 ESC. The animals were handled humanely, kept in plastic suspended cages, and placed in a well-ventilated and hygienic rat house under suitable conditions of room temperature (27 ± 2 °C) and humidity. They were given food and water ad libitum and subjected to a natural photoperiod of 12 h light and 12 h dark cycle. The animals were allowed two weeks of acclimatization before the commencement of all animal model experiments in the study.

All tumor implantations, blood collections, and sacrifices were performed under anesthesia, all efforts being made to minimize suffering.

The study as presented was approved by the Local Ethics Committee of Privolzhsky Research Medical University, Russian Federation (Protocol No. 2 from 20 February 2016).

### 2.4. Biological Activity In Vitro

Biological activity in vitro was studied using blood stabilized with sodium citrate. Erythrocytes were washed twice with 0.9% NaCl by centrifugation for 10 min at 1600 g. Solutions of 0.01% BDP and its mixtures with cytostatics were added to the whole blood (1 mL) at the doses of 2, 5, and 10 µg per mL. The intensity of lipid peroxidation (LPO) was estimated by the MDA level in plasma and erythrocytes in accordance with the methods by Uchiyama and Mihara [28]. Superoxide dismutase activity (EC 1.15.1.1) was measured in erythrocytes using inhibition of adrenaline auto-oxidation [29]. Catalase activity (EC 1.11.1.6) was determined by spectrophotometry based on the decomposition of hydrogen peroxide by the catalase [30]. Glutathione reductase activity (EC 1.8.1.7) was studied by spectrophotometry based on the oxidized glutathione reduction [31]. The activity of glucose-6-phosphate dehydrogenase (EC 1.1.1.49) was determined in hemolysate of erythrocytes by spectrophotometry based on glucose-6-phosphate oxidation to the phosphoglucolactone with the formation of reduced nicotinamide adenine dinucleotide phosphate (NADPH) [32]. The energy metabolism in erythrocytes was studied using the catalytic activity of LDH (LDH, EC 1.1.1.27) in the direct (LDH_direct_, substrate—50 mM sodium lactate) and reverse (LDH_reverse_, substrate—23 mM sodium pyruvate) reactions [33]. The specific activity of the enzymes was calculated from the protein concentration analyzed by the modified Lowry method [34].

### 2.5. Biological Activity In Vivo

Transplanted Ehrlich ascites carcinoma (EAC) was studied in the experiment on mice. 1 mL of the cells of EAC containing 1 × 10^7^ cells was transplanted into the abdominal cavity of a mouse. On day six after transplantation, the resulting ascites liquid was collected. The cells were precipitated at 400 g, thrice washed by 0.15 M NaCl solution, and transplanted into the abdominal cavity of another mouse. Tumor cells were transplanted 2–3 times for cell culture recovery and standardization. The control tumor carrier mouse was prepared after the last transplantation and on day six after transplantation, its cells were used in the experiment. The tumor cells were thrice washed in saline and transplanted into the abdominal cavity of the experimental mice (0.8 mL, 1 × 10^7^ cells∙mL^−1^).

### 2.6. Experimental Protocol

Male albino mice were divided into 6 groups, 10 animals (*n* = 10) in each group, (Table 2).

On day 11, all the mice were sacrificed by decapitation under anesthesia (Zoletil 60 µg∙kg^−1^ “Virbac Sante Animale”, Vauvert, France; Xyla 6 mg∙kg^−1^, Interchemie, Venray, The Netherlands), ascites fluid being collected from the peritoneal cavity. The volume of the fluid was measured using a graduated centrifuge tube.

The cytological analysis of the cells in ascites liquid was made according to Pappenheim’s method with azure-eosin staining and fixation with a 10% formalin solution [35].

The volume of drug administration was 0.2 mL containing 1.5 mg of BDP as its sodium salt. The dose of 5-FU and HS was 0.32 mg per mouse.

Compositions of the used dosage forms are showed in Table 3.

### 2.7. Statistical Analysis

The results obtained in the present study were represented as means ± standard error and were processed using the software Statistica 6.0 (StatSoft Inc., Tulsa, OK, USA).

## 3. Results and Discussion

Biochemical indexes of BDP as a potential pharmaceutical active ingredient isolated from birch bark and then chemically modified had not been studied before, unlike the traditionally used cytostatics. The present study evaluated the effect of BDP and its combination with cytostatics using the biochemical parameters of nontumor rat blood to obtain a response to the biological system.

We studied the dose-dependent influence of BDP on enzyme antioxidant protection by the SOD, catalase, glutathione reductase, and glucose-6-phosphate dehydrogenase activity using nontumor rat blood (Table 4).

Betulin-3,28-diphosphate was shown to lead to similar changes of SOD, glutathione reductase, glucose-6-phosphate dehydrogenase, and catalase activity when using data as percentage of control. Further, we studied the antioxidant enzyme activity under the action of the combination drugs using the SOD activity only.

**Figure 2:**
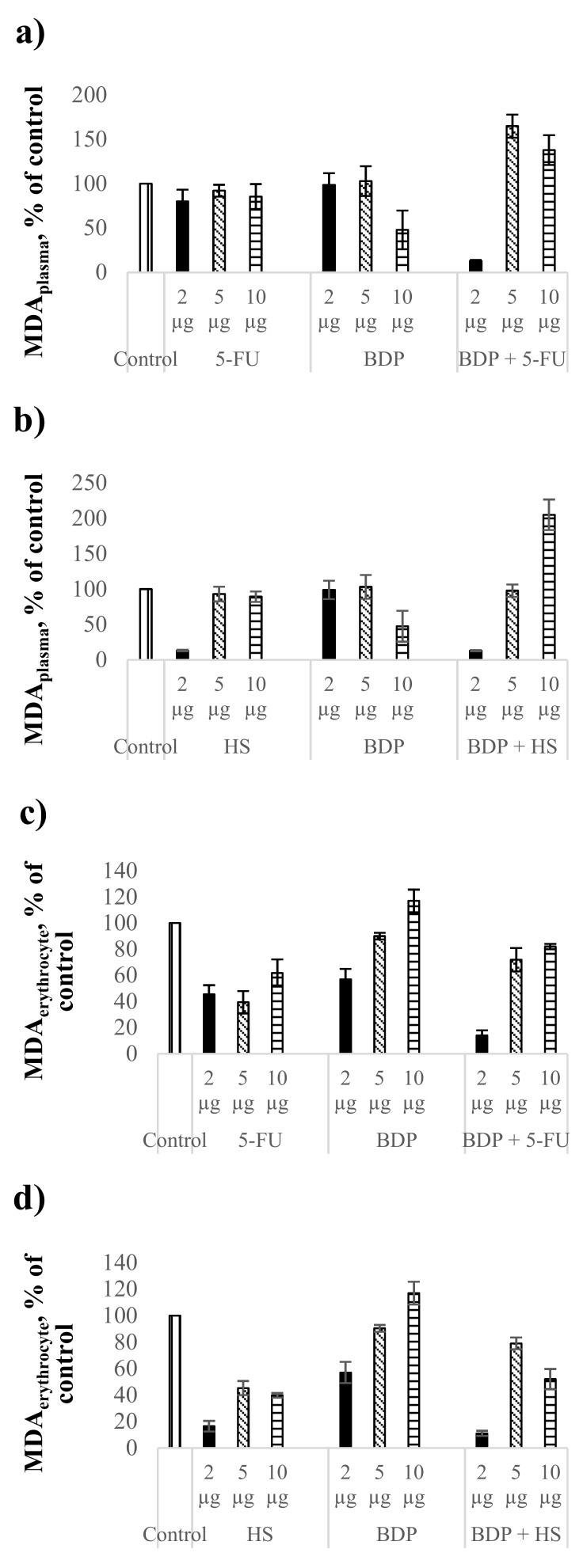

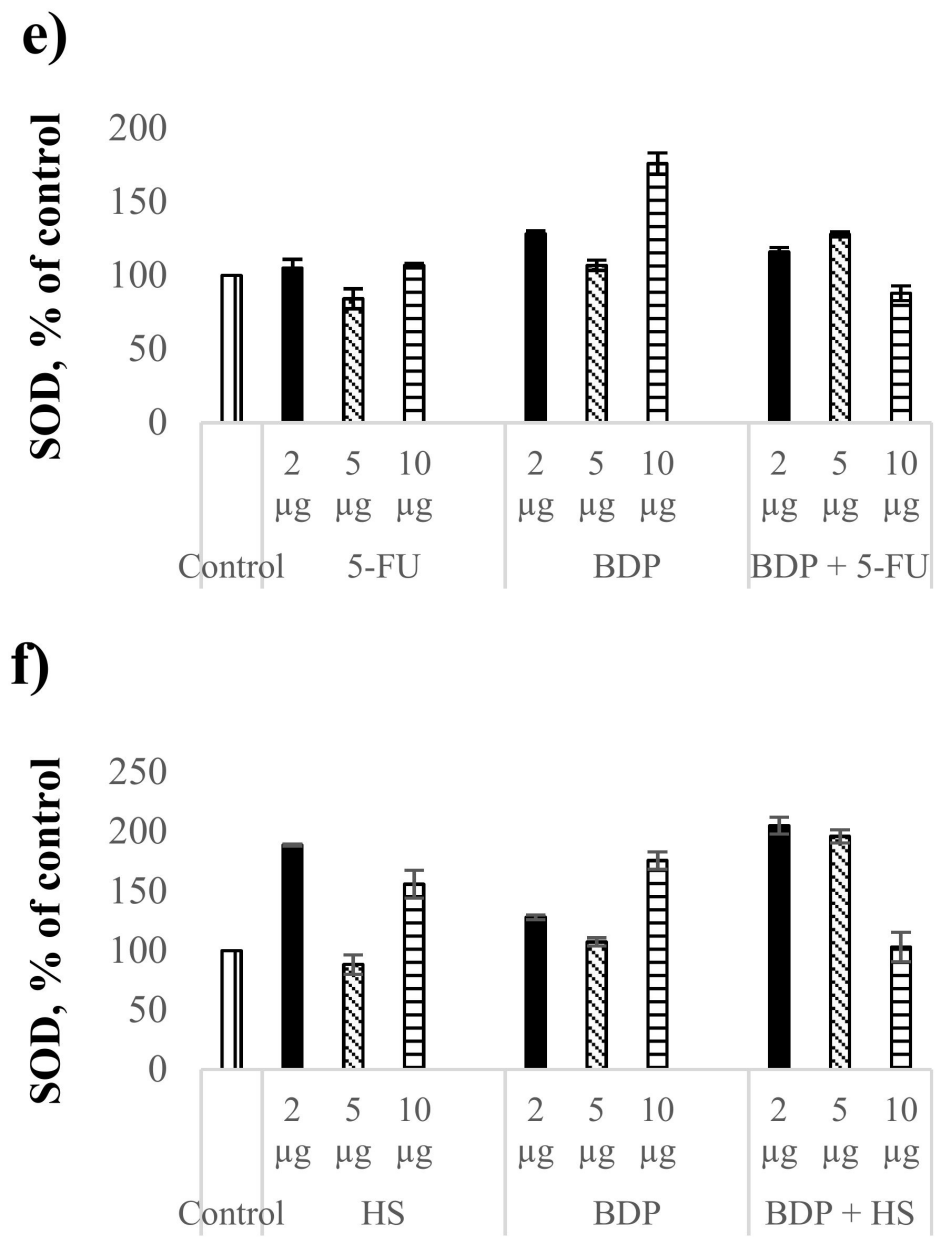
Biochemical indexes under the action of cytostatics and combination drugs at the doses of 2, 5, and 10 μg∙mL^−1^: MDA level in plasma (**a**,**b**) and erythrocytes (**c**,**d**), superoxide dismutase activity (**e**,**f**) as % of control. Biochemical indexes values are taken as 100%: malonic dialdehyde (MDA)_plasma_—0.81433 µmol/L; MDA_erythrocyte_—5.977 µmol/L; SOD—996.95 Ru/mg protein.

The MDA level as the biochemical index of lipid peroxidation intensity was estimated in comparison with the control healthy rat blood without treatment. The action by 5-FU at the dose of 2 μg∙mL^−1^ led to the MDA level decrease by 20% in plasma (Figure 2a, Table 5), but BDP did not reduce the MDA level in plasma. The combination drug BDP + 5-FU showed a pronounced decrease of MDA level by 87 ± 1% in contrast to pure BDP and 5-FU. The fact indicates a high antioxidant effect of BDP + 5-FU drug (Figure 2a).

Hydrazine sulfate showed a decrease of the MDA level in plasma by 87 ± 1%, both pure HS and BDP + HS drugs, at the dose of 2 μg∙mL^−1^, exclusively, while a high antioxidant effect at the doses of 5 and 10 μg∙mL^−1^ was not detected (Figure 2b, Table 5).

Cytostatics showed a stronger antioxidant activity in erythrocytes than in plasma. The MDA level in plasma under the action of 5-FU and HS decreased by 38–61% and 60–84%, respectively (Figure 2c,d, Table 5). Betulin-3,28-diphosphate influenced the MDA level in erythrocytes at the dose of 2 μg∙mL^−1^ only, with the MDA level decreasing by 43 ± 8%. The combination drugs BDP + 5-FU and BDP + HS produced the synergetic effect, reducing the MDA level in erythrocytes by 86 ± 4% and 89 ± 2%, respectively (Figure 2c,d).

In conclusion, all the studied combination drugs of BDP with cytostatics have a significant impact on lipid peroxidation processes both in plasma and erythrocytes.

There was observed a dose-dependent effect on SOD activity of BDP and the combination drug BDP + 5-FU. The SOD activity under the BDP action increased by 28% at the dose of 2 µg·mL^−1^ and by 76% at the dose of 10 µg·mL^−1^ compared to the control, while the combination drug BDP + 5-FU increased the SOD activity by 16% at the dose of 2 µg·mL^−1^ and by 28% at the dose of 5 µg·mL^−1^ (Figure 2e,f, Table 5).

The effect of hydrazine sulfate on the SOD activity compared to BDP + 5-FU was found to be significantly greater both under the action of pure HS and under the action of the combination drug BDP + HS. The SOD activity increased twice in case of combination drug BDP + HS (Figure 2f, Table 5).

In general, it can be noted that the antioxidant properties of BDP and its combination drugs are greater in the enzyme processes than the antioxidant activity in LPO processes (Figure 2a,b, Table 5). Lipid peroxidation estimated by the MDA level has a dysregulated effect on cell growth, proliferation and changes in DNA formation that is due to direct and indirect cytotoxic effects. Non-enzymatic protection against oxidative stress by combination drugs treatment suggests the reduction of lipid peroxidation caused by reactive oxygen species (superoxide, hydrogen peroxide, hydroxyl free radicals) and by nitrogen oxidation products involved in carcinogenesis both at the stages of cancer induction and at its apoptosis-necrotic stages and levels of gene mutations [36,37]. The increase of SOD activity is associated with the activation of genes encoding the family of cytoprotective proteins including superoxide dismutase. Since the oxidative damage of cells plays an important role in the mechanism of carcinogenesis, the data on high antioxidant enzyme protection obtained in the present work are a good predictor of the antitumor activity of the combination drugs.

**Figure 3:**
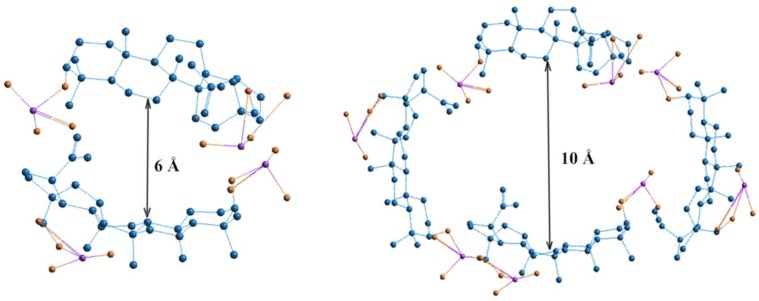
Geometric visualization of BDP as a component of inclusion complexes formed by two and four molecules. Quantum-chemical calculations were provided by HyperChem 8.0, Hypercube Inc, Gainesville, FL, USA (semiempirical method AM1). Size of the void between BDP molecules is 6 Å for dimer and 10 Å for tetramer [27].

Dose-dependent effects of studied drugs can be explained by considering their redox potential. The cytostatics such as 5-FU and HS have different antioxidant potentials, which is most fully reflected at the dose of 2 μg. Hydrazine sulfate has the more optimal redox potential in comparison with 5-FU and exhibits stronger antioxidant properties in both aqueous and lipid media. At higher doses, biologically active substances having greater antioxidant activity, such as ascorbic acid and phenols, tend to have a greater pro-antioxidant effect. The pro-antioxidant effect of cytostatics was manifested in combination with BDP, which is reflected by MDA level increase combined with cytostatic + BDP at the doses of 5 and 10 μg. The sharp decrease in MDA level is probably due to the role of BDP dimers or tetramers as a vector for the drug delivery of toxic cytostatics by forming the inclusion complex BDP-cytostatic at a low dose, whereas a part of the cytostatic is not involved in inclusion complexes BDP + HS at high doses of drug. Earlier, the formation of complexes BDP with other amines, trisamine and dopamine, was shown by thermodynamic methods (Figure 3).

The LDH activity in the direct and reverse reactions grew under the influence of pure 5-FU and HS at all the doses (Table 6), indicating an increase in the energy metabolism in erythrocytes. In case of 2 µg·mL^−1^ dose, LDH_reverse_ activity increased under the influence of pure 5-FU and HS by 101 ± 9% and 297 ± 3%, respectively, in comparison with the control. The pure BDP at the same dose increased the LDH_reverse_ activity by 54 ± 4%, while the LDH_direct_ activity did not depend on BDP compared to the control.

The LDH_direct_ and LDH_reverse_ activities changed insignificantly when using the combination drug BDP + 5-FU, while the LDH_direct_ and LDH_reverse_ activities were above the control group by 23% and 195%, respectively, at the dose of 2 µg·mL^−1^.

Thus, the combination drugs of BDP with cytostatics generally influence the LDH_reverse_ activity. The effect is most strongly manifested in the case of the combination drug of BDP with hydrazine sulfate.

The rise of LDH_reverse_ activity characterizes the increase in the lactic acid content, which is formed mainly by M-subunits of LDH (LDH_reverse_) and indicates the predominance of anaerobic processes in erythrocytes.

The results are probably related to the Pasteur’s effect, which combines the processes of glycolysis and respiration that is especially significant for tumor cells [38]. The glycolytic enzymes and mitochondrial systems of cancer cells are not different from normal ones, although the ways of combining the processes of glycolysis and respiration in normal and tumor cells are obviously different. For this reason, tumor cells in contrast to normal cells in the breathing processes accumulate a significant amount of lactate, even under complete oxygenation and a high respiration rate. One of the reasons for the lactate excess is the deficiency of cytoplasmic glycerophosphate dehydrogenase in tumor cells, therefore the glycerophosphate shuttle mechanism cannot occur effectively. As tumor cells are unable to oxidize NADH by a mitochondrial way, they reoxidize NADH using pyruvate by lactate dehydrogenase; in this case, lactate accumulates in aerobic conditions, although the tricarboxylic acid cycle and the electron transport chain have a normal rate of biochemical reactions typical for aerobic conditions in tumor cells. Accordingly, the shift toward the reverse reaction is favorable for tumor cells.

There are many discursions about the Warburg effect too. Now, it has been established that the energy metabolism of tumor cells is determined by the balance between glycolysis and oxidative phosphorylation, whereas the effect of pure Warburg glycolysis in tumor cells is disputed [39,40,41,42,43]. With the ability of drugs to activate energy metabolism in tumor cells, both the glycolysis and oxidative phosphorylation (the increase in the LDH_direct_ and LDH_reverse_ activity) can be only explained as the effectiveness of the drugs studied for survival.

We believe that the metabolic plasticity observed under the action by BDP + HS in vitro using erythrocytes of nontumor rats may have an impact on tumor physiology in vivo. Therefore, these effects can be used as the elements of metabolic therapy.

### The Characteristics of the Betulin-3,28-diphosphate Dosage Form in the Treatment of Ehrlich Carcinoma in Mice

The treatment of Ehrlich carcinoma in mice by combination drugs BDP + 5-FU and BDP + HS showed improvement in objective indicators of the state of animals (survival, body weight, wool, behavior, reduced cardiotoxicity). All the animals in the experimental groups survived, while not more than 80 ± 5% of the animals survived in control groups EAC-5-FU and EAC-HS.

The ascites fluid in the peritoneal cavity was carefully collected into plastic tubes from a hole opened with a syringe at abdominal site, and then from the opened abdominal cavity with scissors under anesthesia (Zoletil, 60 µg·kg^−1^, Xyla, 6 mg·kg^−1^) on the 11th day and then kept in an ice-cold bath.

The effects of cytostatics and combination drugs BDP + HS and BDP + 5-FU on the accumulation of ascites fluid were examined on day 11 (Table 7). The administration of a daily repeated dose of 0.2 mL per mouse (1.5 mg of BDP and 0.32 mg of cytostatics per mouse) for 11 days showed no abnormal behavioral responses. Mice treated only with cytostatics displayed significantly deteriorated health status (wool state, body weight, and behavior) than those in the control group or EAC-(BDP + 5-FU) and EAC-(BDP + HS) groups.

The fluid volume in the control group was negligible, but the volume of the fluid in the EAC-control group was very large. The volumes of the ascites fluid in EAC-5-FU and EAC-HS groups were one and half less than that of the EAC-control group. In contrast, the ascites fluid volumes of combination drugs groups were thrice less than volumes of EAC-control group on the 11th day.

Histological analysis has not shown any significant changes in the heart tissues in the EAC-(BDP + 5-FU) and EAC-(BDP + HS) groups, whereas dystrophic changes and the loss of transverse striation in some muscle fibers were noted in cardiomyocytes of the control-EAC group.

The concentration of neutrophil and tumor cells under treatment by BDP + 5-FU and BDP + HS combination drugs were significantly decreased (Table 7).

The cytological study of ascetic liquid after the treatment by the combination drugs BDP + HS and BDP + 5-FU showed no tumor cells, whereas the tumor cells were found in control groups EAC-5-FU and EAC-HS.

The important advantages of phosphate-containing molecules are their high ability to interact with amines, amino acids, amino-and NH-groups of proteins [44], and according to Cotton et al. [45], it is a crucial property in life processes. The nature of the interaction of phosphate with nitrogen-containing fragments is varied and includes both covalent and hydrogen bonds as well salt formation as complexation due to nonspecific noncovalent binding [46].

The formation of BDP salt complexes with amines is expected to improve its bioavailability.

## 4. Conclusions

The work has estimated the synergetic effects of cytostatic 5-FU and monoamine oxidase inhibitor HS on oxidative stress decrease and energy metabolism improvement in the presence of BDP in rat blood.

A dose-dependent effect was revealed when combination drugs of cytostatics with BDP were tested by in vitro experiments on rat blood. The study demonstrated the possibility of reducing the dose (up to five times) of highly toxic cytostatics in the proposed pharmaceutical composition.

The treatment of transplanted Ehrlich ascites carcinoma in mice with BDP in combination with cytostatics 5-FU and HS led to an improvement in the animals’ state. The increased survival rate, improvement in body weight, wool, and behavior, decrease in cardiotoxicity, the tumor cell concentrations, and ascites fluid volume were observed in contrast to the control groups treated only by cytostatics.

Thus, the findings enable the use the combination drugs BDP with cytostatics such as 5-FU and HS to optimize polychemotherapy of Ehrlich ascites carcinoma. The decrease of highly toxic cytostatics doses during their single administration creates the prerequisites for using these compositions in palliative drug therapy.

## Figures and Tables

**Table 1:** Carbon-13 nuclear magnetic resonance (^13^C-NMR) spectral data of betulin-3,28-diphosphate.

C	*δ* (ppm)	C	*δ* (ppm)	C	*δ* (ppm)	C	*δ* (ppm)	C	*δ* (ppm)	C	*δ* (ppm)
C1	38.71	C2	28.27	C3	83.32	C4	38.93	C5	55.25	C6	18.36
C7	36.93	C8	40.84	C9	50.04	C10	37.42	C11	20.77	C12	25.18
C13	37.10	C14	42.66	C15	26.92	C16	29.31	C17	47.11	C18	47.62
C19	48.46	C20	150.31	C21	29.46	C22	34.13	C23	28.53	C24	16.06
C25	15.27	C26	16.51	C27	14.92	C28	63.60	C29	110.28	C30	19.19

C: number of carbon atom, δ (ppm): chemical shift.

**Table 2:** The characteristic of animal experimental groups.

Control group	Intact mice
Control EAC group	EAC-bearing mice
EAC-5-FU group	EAC-bearing mice treated with 5-fluorouracil (5-FU) i.p. once a day
EAC-HS group	EAC-bearing mice treated with hydrazine sulfate (HS) i.p. once a day
EAC-(BDP + 5-FU) group	EAC-bearing mice treated with the combination drug of BDP with 5-fluorouracil (5-FU) i.p. once a day
EAC-(BDP + HS) group	EAC-bearing mice treated with the combination drug of BDP with hydrazine sulfate (HS) i.p. once a day

EAC: Ehrlich ascites carcinoma, BDP: betulin-3,28-diphosphate, i.p.: intraperitoneally.

**Table 3:** Drugs Compositions.

Group	Cytostatic *(mg)	BDP (mg)
EAC-HS	HS—48	-
EAC-5-FU	5-FU—48	-
EAC-(BDP + HS)	HS—64	200
EAC-(BDP + 5-FU)	5-FU—64	200

* Water up to 32 mL.

**Table 4:** Dose-dependent effects of BDP on biochemical indexes of nontumor rat blood.

	SOD (% of Control)	Catalase (% of Control)	Glutathione Reductase (% of Control)	Glucose-6-phosphate Dehydrogenase (% of Control)
BDP 2 μg	135 ± 2	136 ± 3	134 ± 3	132 ± 3
BDP 5 μg	105 ± 3	102 ± 4	103 ± 3	127 ± 4
BDP 10 μg	192 ± 5	204 ± 7	183 ± 2	237 ± 2

Number of replication of experiments was equal to 3. Biochemical indexes values are taken as 100%; superoxide dismutase (SOD)—845.58 Ru/mg protein, catalase—31.35 Ru/mg protein; glutathione reductase—91.29 nmol nicotinamide adenine dinucleotide phosphate (NADH)/min/mg protein, glucose-6-phosphate dehydrogenase—52.37 nmol NADPH/min/mg protein.

**Table 5:** Malonic dialdehyde level in plasma and erythrocytes, superoxide dismutase activity (% of control) under the action of 5-FU, HS, BDP, BDP + 5-FU and BDP + HS at the doses of 2, 5, and 10 μg∙mL^−1 a^.

	Dose (µg·mL^−1^)	MDA_plasma_ (% of Control)	MDA_erythrocyte_ (% of Control)	SOD (% of Control)
BDP	2	99 ± 13	57 ± 8	128 ± 2
	5	103 ± 17	90 ± 3	107 ± 4
	10	48 ± 22	117 ± 9	176 ± 7
5-FU	2	80 ± 13	45 ± 7	105 ± 6
	5	92 ± 7	39 ± 9	84 ± 7
	10	86 ± 14	62 ± 10	107 ± 2
BDP + 5-FU	2	13 ± 1	14 ± 4	116 ± 3
	5	165 ± 13	72 ± 9	128 ± 2
	10	138 ± 17	82 ± 2	88 ± 5
HS	2	13 ± 1	16 ± 4	189 ± 1
	5	93 ± 10	45 ± 5	88 ± 8
	10	89 ± 7	40 ± 2	156 ± 12
BDP + HS	2	13 ± 1	11 ± 2	205 ± 7
	5	98 ± 9	79 ± 4	196 ± 6
	10	205 ± 22	52 ± 8	103 ± 13

^a^ Number of replication of experiments was equal to 3.

**Table 6:** Lactate dehydrogenase activity (% of control) in direct and reverse reactions under the action of 5-FU, HS, BDP, BDP + 5-FU, and BDP + HS at the doses of 2, 5 and 10 μg∙mL^−1^.

		Index, % of Control	
	Dose (µg·mL^−1^)	LDH_direct_	LDH_reverse_
BDP	2	99 ± 7	154 ± 4
	5	130 ± 3	98 ± 21
	10	134 ± 15	71 ± 3
5-FU	2	124 ± 8	201 ± 9
	5	102 ± 16	85 ± 5
	10	115 ± 4	163 ± 3
BDP + 5-FU	2	100 ± 9	106 ± 5
	5	102 ± 10	112 ± 11
	10	131 ± 11	105 ± 4
HS	2	124 ± 10	397 ± 3
	5	136 ± 8	97 ± 13
	10	164 ± 6	134 ± 5
BDP + HS	2	123 ± 3	295 ± 21
	5	132 ± 2	158 ± 3
	10	148 ± 1	227 ± 3

Number of experiment replications was equal to 3. Biochemical indexes are taken as 100%: LDH_direct_—60.87 nmol NADH/min/mg protein; LDH_reverse_—125.58 nmol NADH/min/mg protein. LDH: lactate dehydrogenase.

**Table 7:** The effects of 5-FU, HS, and the combination drugs of BDP + 5-FU and BDP + HS on the biological parameters of EAC-bearing mice on the 11th day ^a^.

	Survival	Body Weight		Ascites Fluid (mL) on the 10th Day	Tumour Cells ^b^	Neutrophil ^c^
		Day 1	Day 11			
Control EAC	3/10	23.1 ± 0.6	29.2 ± 0.5	6.0 ± 0.3	++	+++
EAC-5-FU	8/10	21.8 ± 0.7	26.3 ± 1.1	4.0 ± 0.5	+	++
EAC-HS	6/10	22.3 ± 0.6	27.1 ± 0.9	4.5 ± 0.4	+	++
EAC-(BDP+5-FU)	10/10	22.5 ± 0.5	25.3 ± 0.5	2.3 ± 0.6	-	+
EAC-(BDP+HS)	10/10	23.0 ± 0.5	26.2 ± 0.7	2.6 ± 0.4	-	+
Control	10/10	24.5 ± 0.4	25.1 ± 0.3	0	-	+

^a^ Values are means ± standard deviation (*n* = 10). EAC: Ehrlich ascites carcinoma. ^b^ - negligible; + means tumor cells content more than 5%; ++ tumor cells content more than 10%. ^c^ + neutrophil content corresponding to the neutrophil content of a healthy animal; ++ neutrophil content that twice more than the neutrophil content of a healthy animal; +++ neutrophil content that thrice more than the neutrophil content of a healthy animal.

## Abbreviations

BDP	betulin-3,28-diphosphate
HS	hydrazine sulfate
5-FU	5-fluorouracil
BDP + HS and BDP + 5-FU	combination drugs
SOD	superoxide dismutase
MDA	malonic dialdehyde
LDH	lactate dehydrogenase
LPO	lipid peroxidation
MAO	monoamine oxidase
EAC	Ehrlich ascites carcinoma
